# Rationally Trust, but Emotionally? The Roles of Cognitive and Affective Trust in Consumer Imitation Intention Toward Influencers in Short-Form Live Commerce

**DOI:** 10.3390/bs16091641

**Published:** 2026-09-14

**Authors:** Yanxi Chen, Byoungsoo Kim

**Affiliations:** School of Business, Yeungnam University, 280 Daehakro, Gyeongsansi 38541, Republic of Korea

**Keywords:** live commerce, short-form video, influencer characteristics, content characteristics, imitation intention, cognitive trust, affective trust

## Abstract

This study examines how influencer characteristics and short-form video content characteristics shape consumers’ imitation intention toward influencers in short-form live commerce. Drawing on the stimulus–organism–response (S-O-R) framework and social learning theory, this study conceptualizes cognitive trust and affective trust as two internal psychological states linking external stimuli to imitation intention. Imitation intention is defined as consumers’ willingness to regard influencers as behavioral reference models and to adopt or follow their consumption choices, suggestions, and recommendations. Survey data were collected from 306 Chinese consumers of TikTok, Kuaishou, and Xiaohongshu and analyzed using partial least squares structural equation modeling (PLS-SEM). The results show that ethics, homophily, authenticity, and entertainment significantly enhance both cognitive and affective trust, whereas originality significantly increases only cognitive trust and social attractiveness has no significant effect on either trust dimension. Cognitive trust also positively influences affective trust. Both trust dimensions significantly increase consumers’ imitation intention, with affective trust exerting the stronger effect. The mediation analysis further reveals both differentiated parallel and sequential trust pathways: cognitive trust mediates five of the six antecedent relationships, affective trust mediates four, and sequential mediation through cognitive trust and affective trust is significant for ethics, homophily, authenticity, originality, and entertainment. These findings show that trust operates through both distinct and interrelated mechanisms in translating influencer- and content-related cues into imitation intention.

## 1. Introduction

The convergence of short-form video and live commerce represents one of the most consequential transformations in contemporary digital retail (B. Kim et al., 2023; Ming et al., 2021). Fueled by algorithmic recommendation systems and the rapid expansion of creator-led entrepreneurship, platforms such as TikTok, Kuaishou, and Xiaohongshu have fused short-form entertainment with real-time shopping into an increasingly seamless consumer experience. By the end of 2024, China’s short-form video user base had exceeded one billion, while live commerce accounted for a growing proportion of total e-commerce transactions (CNNIC, 2025). This shift suggests that short-form video is no longer merely a promotional channel; rather, it has become a foundational mechanism through which customer attention is cultivated, commercial relationships are formed, and transactional behavior is activated. Within this evolving ecosystem, influencers occupy a structurally distinctive position. They attract and retain audiences through short-form content, such as daily vlogs, beauty tutorials, product demonstrations, and niche informational content, and subsequently monetize those audiences through live commerce. As customers repeatedly engage with an influencer’s content, they are likely to develop both cognitive evaluations and emotional attachments toward the influencer (Li et al., 2025). From a relational perspective, influencers aim to establish deeper psychological connections with their followers in order to build long-term relationships (Belanche et al., 2021). Therefore, influencers’ characteristics are crucial factors in attracting consumers (Walter et al., 2025). For influencers on short-form video platforms, the video content they create is also an important factor influencing consumers (Manic, 2024). Accordingly, this study selects antecedents of trust from two dimensions: influencer characteristics and content characteristics. At the influencer-characteristics level, social attractiveness, ethics, and homophily respectively reflect consumers’ evaluations of influencers’ potential social interaction partner, moral reliability, and similarity, and these factors help consumers determine whether influencers are trustworthy. At the content-characteristics level, authenticity, originality, and entertainment respectively reflect consumers’ evaluations of the authenticity and credibility, uniqueness, and pleasurable experience of the content provided by influencers, and further influence consumers’ cognitive and affective responses toward influencers.

Trust plays a critical role in consumers’ decision-making in live commerce, where the credibility of influencers is often difficult to verify and consumers must make decisions under uncertainty, time pressure, and interpersonal influence (Chen et al., 2024; Shamim & Islam, 2022). Trust therefore helps reduce uncertainty and guides consumers’ subsequent behavioral responses (B. Kim et al., 2025). Rather than treating trust as a unidimensional construct, this study adopts McAllister’s (1995) distinction between cognition-based and affective trust. Cognitive trust is grounded in rational assessments of an influencer’s competence, reliability, and consistency, whereas affective trust arises from perceived care, emotional bonds, and relational closeness. Importantly, these two forms of trust are not necessarily independent, as rational evaluations of an influencer’s reliability may provide a foundation for the development of affective trust. Building on McAllister (1995), this study further examines how cognitive trust contributes to the development of affective trust and how these two forms of trust subsequently influence consumers’ imitation intention.

These relational bonds may then be activated in live commerce settings, shaping not only customers’ purchase decisions but also their willingness to emulate the influencer’s preferences, judgments, and consumption-related behaviors. Previous research on influencer marketing and live commerce has widely used purchase intention as an important behavioral outcome variable, providing an important foundation for understanding how influencer characteristics and content characteristics promote consumers’ purchase decisions. However, purchase intention mainly reflects consumers’ willingness to purchase a specific product and is therefore essentially a product- and transaction-centered behavioral outcome. This outcome variable may not fully capture the broader social influence that influencers exert on consumers (Sokolova & Kefi, 2020). Particularly on short-video platforms, repeated content exposure and real-time interactions enable influencers to cultivate ongoing relationships with consumers beyond individual transactions. In this context, consumers not only evaluate whether to purchase a particular product but may also regard influencers as behavioral reference models and align their own consumption choices with the behaviors displayed or advocated by influencers (Ampornklinkaew, 2025). From this perspective, imitation intention differs from purchase intention in that it reflects consumers’ tendency to regard influencers as behavioral reference models and their willingness to adopt or follow influencers’ consumption choices, suggestions, and recommendations.

From the perspective of social learning theory, individuals can develop new behavioral tendencies by observing the behaviors of important others and regarding them as role models (Bandura, 1977). Therefore, examining imitation intention allows this study to move beyond a transaction-centered research perspective and further investigate the social learning process through which influencers become behavioral reference models for consumers. Specifically, this study focuses on whether consumers’ cognitive trust and affective trust in influencers can lead them to regard influencers as trustworthy behavioral reference models and further strengthen their willingness to follow influencers’ consumption choices and recommendations. Therefore, in short-video-based live commerce, imitation intention constitutes a theoretically meaningful and contextually appropriate outcome.

To address this gap, this study adopts the S-O-R framework to organize the overall research model. This study does not aim to extend the S-O-R theory itself, but rather employs it as an overarching analytical framework to systematically explain the process of “perceptions of influencer and content characteristics—consumer trust—imitation intention.” Building upon this framework, this study further integrates the dual-trust perspective and social learning theory to explain the formation relationship between cognitive trust and affective trust, as well as the specific psychological mechanism through which trust is further transformed into consumers’ imitation intention. Accordingly, this study addresses the following research questions:

RQ1. How do influencer-related and content-related characteristics shape consumers’ cognitive and affective trust in influencers in live commerce?

RQ2. How does cognitive trust contribute to the development of affective trust in influencer–consumer relationships?

RQ3. How do cognitive and affective trust translate into consumers’ imitation intention toward influencers in live commerce?

This study makes two contributions to the literature. First, this study moves beyond conventional transaction-oriented outcomes in live-commerce research by positioning imitation intention as a distinct influencer-directed behavioral response. By integrating social learning theory, this study extends the understanding of consumer behavior in live commerce from “how influencers facilitate product transactions” to “how influencers become trustworthy behavioral reference models for consumers and induce behavioral imitation,” thereby revealing the social learning mechanism in influencer marketing that goes beyond mere transactional responses. Second, this study further deepens the understanding of the formation and development of consumer trust in the context of live commerce. Rather than treating trust as a single construct or simply viewing cognitive trust and affective trust as parallel psychological states, this study distinguishes between cognitive trust and affective trust and examines their different formation paths from the two dimensions of influencer characteristics and content characteristics. At the same time, by considering how cognitive trust contributes to the development of affective trust, this study further reveals how consumers’ rational assessments of an influencer’s reliability can facilitate the development of affective trust and ultimately influence their imitation intention.

## 2. Theoretical Background and Research Model

### 2.1. The S-O-R Framework in Live Commerce

The S-O-R framework proposes that environmental stimuli influence individuals’ internal cognitive and affective states, which subsequently shape behavioral responses (Mehrabian & Russell, 1974). By incorporating internal psychological states between external stimuli and behavioral outcomes, S-O-R provides a useful framework for explaining how contextual cues are translated into consumer responses. Previous studies have applied this framework in e-commerce and live commerce to examine how platform-, streamer-, and content-related cues influence consumers’ psychological states and subsequent behavioral intentions (Ming et al., 2021; Yang et al., 2024; Liang et al., 2026). Accordingly, this study employs S-O-R as an organizing framework rather than seeking to extend the theory itself. Specifically, influencer- and content-related characteristics are conceptualized as stimuli, cognitive and affective trust as organismic states, and imitation intention as the behavioral response. To further explain the relationships among these psychological states and behavioral outcomes, the study additionally draws on the dual-trust perspective and social learning theory.

### 2.2. Influencer-Characteristics and Content-Characteristics

Influencers play a crucial role in social media and can influence the ways their followers or audiences think and behave (Yenita & Cintalia, 2023). Therefore, influencers’ characteristics are important factors in exploring consumer behavior. Previous research has shown that influencer-related attributes, such as social attractiveness, similarity, expertise, and credibility, play an important role in shaping consumers’ perceptions of social media influencers and their responses (Le & Hancer, 2021). Every piece of content shared by influencers can also indirectly encourage consumers to imitate them (Yenita & Cintalia, 2023). At the same time, recent research emphasizes that social media influencers are essentially content creators, and that the characteristics of influencer-generated video content affect consumers’ perceptions of influencer credibility and their subsequent behavioral responses (Ampornklinkaew, 2025). In particular, appealing and attention-grabbing content can enhance perceptions of influencers’ credibility and expertise, whereas low-quality or unappealing content may undermine their credibility. Based on these two complementary sources of evaluation, this study selects six antecedents of trust. At the influencer level, social attractiveness, ethics, and homophily together constitute three distinct interpersonal judgments: whether the influencer is perceived as socially attractive, morally reliable, and similar to the consumer. At the content level, authenticity, originality, and entertainment determine consumers’ evaluations of whether influencer-generated content is authentic, unique, and enjoyable. These content-related dimensions are particularly important because prior research has shown that authentic content helps influence consumers’ decision-making, while novelty, interest, and enjoyment can shape consumers’ responses to social media content. Taken together, these six factors provide a theoretically coherent and concise representation of two complementary sources of trust formation—that is, evaluations of the influencer as a person and evaluations of the content created by the influencer—rather than attempting to exhaust all possible antecedents of trust.

### 2.3. Cognitive Trust and Affective Trust in Live Commerce

Trust has been conceptualized as both a unidimensional and a multidimensional construct (Lewis & Weigert, 1985). While some scholars regard trust as a general willingness to be vulnerable, others argue that it consists of qualitatively distinct dimensions (McKnight et al., 2002; Rousseau et al., 1998). Among multidimensional approaches, McAllister’s (1995) distinction between cognitive trust and affective trust has been widely adopted and increasingly applied in live commerce research (Chen et al., 2024; Ha et al., 2016).

Cognitive trust and affective trust are regarded as internal organismic states shaped by external stimuli in the live commerce environment and subsequently influencing customers’ behavioral responses (Li et al., 2025). Trust is therefore an appropriate organism variable because it reflects an internal psychological state formed through cognitive and affective processing of live commerce stimuli and links environmental cues to customer responses (Zhang et al., 2022). Cognitive trust is formed through customers’ rational evaluations of an influencer’s competence, reliability, dependability, and professionalism (McAllister, 1995; Mayer et al., 1995). In live commerce, it may stem from the influencer’s product knowledge and the accuracy and specificity of product demonstrations (Wongkitrungrueng & Assarut, 2020), reflecting customers’ judgment that the influencer is competent and dependable. In contrast, affective trust is rooted in emotional bonds and interpersonal care (McAllister, 1995). In live commerce, it develops through sustained real-time interaction, personalized responses, personal disclosure, and expressions of care that foster emotional attachment and relational closeness (Wongkitrungrueng & Assarut, 2020; Sun et al., 2019). Thus, affective trust reflects customers’ emotional security, confidence, and perceived care toward influencers.

### 2.4. Imitation Intention and Social Learning Theory

Ampornklinkaew (2025) also pointed out that imitation intention refers to consumers’ willingness to regard an influencer as a behavioral reference and to adopt or follow the consumption choices, suggestions, and recommendations provided by that influencer. Ampornklinkaew (2025) also pointed out that purchase intention is commonly examined in consumer behavior research because of its role in driving sales, whereas imitation intention focuses more on the influencer, with followers attempting to imitate the influencer’s behavior and regarding the influencer as an idol. This distinction is particularly important in influencer-driven live commerce because influencers not only promote products but also continuously present product choices, usage experiences, and consumption-related preferences to their audiences.

Social learning theory provides a useful theoretical foundation for explaining such imitative behavior. According to social learning theory, individuals can acquire attitudes and behaviors by observing others rather than solely through direct experience (Bandura, 1977). Observational learning typically involves attention, retention, reproduction, and motivation, where reproduction reflects the observer’s physical ability to imitate the model’s behavior, and motivation refers to the willingness to imitate such behavior (Bandura, 1977). In social media environments, such learning is more likely to occur when the observed person is regarded as a meaningful behavioral model, and the emergence of influencers as new actors in the digital domain has strengthened the importance of social learning theory (Muniasari, 2026). Influencers can serve as such role models because consumers repeatedly observe their product evaluations, consumption choices, and recommendations. Previous research has likewise applied social learning theory to explain consumers’ willingness to imitate social media influencers, suggesting that influencer characteristics can affect whether audiences perceive them as suitable role models for behavioral imitation (Le & Hancer, 2021).

In the context of this study, social learning theory helps explain why consumers may move beyond merely evaluating influencers and instead use them as behavioral references. Trust, distrust, influencer credibility, and attitudes toward influencers are key drivers of imitation intention (Belanche et al., 2021). When consumers perceive an influencer as trustworthy and worthy of reliance, the influencer’s consumption-related judgments and recommendations are more likely to be regarded as appropriate for observation and adoption. Therefore, imitation intention represents a behavioral response through which consumers translate their evaluations of influencers into a willingness to follow their consumption choices, suggestions, and recommendations.

## 3. Research Model and Hypotheses

**Figure 1:**
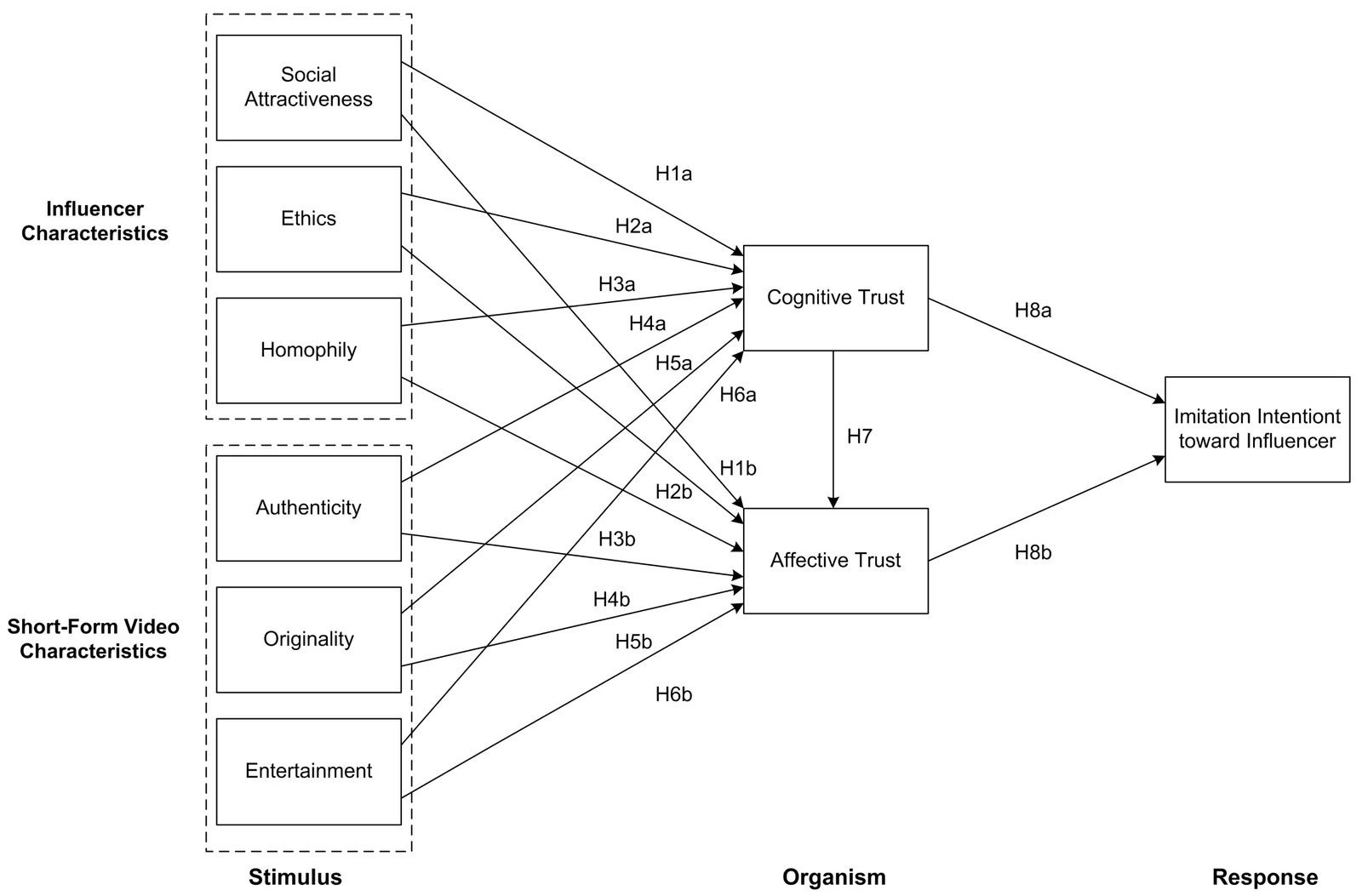
Research model.

This study applies the S-O-R framework to explain consumers’ imitation intention toward influencer in the short-form live commerce context. Stimuli are the observable characteristics of influencers and their short-form video content. Organism states are the internal psychological responses they evoke—specifically, cognitive trust and affective trust. The behavioral response is consumers’ intention to take the influencer as a behavioral reference and to adopt or follow the consumption choices, suggestions, and recommendations presented by the influencer. The research model is depicted in Figure 1.

### 3.1. Influencer Characteristics

#### 3.1.1. Social Attractiveness

Social attractiveness refers to the extent to which a communicator is perceived as likable, charming, and approachable (J. C. McCroskey & McCain, 1974). In the context of live commerce, it captures an influencer’s friendliness and the sense of peer-like intimacy they create with viewers. Social attractiveness was selected because it captures the interpersonal and relational dimension of consumers’ evaluations of influencers, which is particularly salient in live commerce where repeated interaction and communication make social impressions an important basis for trust formation. This perception can be indirectly reflected through observable indicators such as the number of likes, followers, and real-time viewership. Prior research has shown that social attractiveness is an important antecedent of trust in social media influencer contexts (Masuda et al., 2022). Influencers who convey warmth, empathy, and a confident yet approachable self-presentation are more likely to be perceived as socially attractive (Sokolova & Kefi, 2020). Social attractiveness is expected to enhance cognitive trust because likability serves as a peripheral but meaningful informational cue. Viewers may infer that a socially attractive streamer possesses the communication skills, credibility, and professional competence necessary to maintain such a position. These inferences provide rational grounds for evaluating the streamer as a reliable source of purchase-related information (McAllister, 1995; Shoenberger & Kim, 2023). At the same time, social attractiveness is also expected to strengthen affective trust, as likability naturally evokes interpersonal liking and positive emotional responses toward the influencer.

**H1a.**  *Social attractiveness is positively associated with cognitive trust.*

**H1b.**  *Social attractiveness is positively associated with affective trust.*

#### 3.1.2. Ethics

Influencer ethics refers to the extent to which an influencer adheres to moral norms in the process of content creation (Aboelenien et al., 2023; M. Kim & Baek, 2025). In live commerce, ethical behavior is reflected in practices such as clearly disclosing sponsored content, providing truthful product descriptions that acknowledge both strengths and limitations. Ethics was selected because live commerce involves strong commercial persuasion and information asymmetry, making consumers particularly sensitive to whether influencers communicate honestly, transparently, and with consideration for consumers’ interests (Wellman et al., 2020). Ethics is expected to enhance cognitive trust because ethical conduct serves as a diagnostic cue of the streamer’s integrity. When influencers communicate transparently and honestly, consumers have rational grounds to believe that the recommendations are based on genuine evaluations rather than purely commercial intentions. Ethics is also expected to strengthen affective trust. Perceived moral integrity signals that the streamer cares about consumers’ welfare, rather than treating them merely as targets of persuasion (Wei et al., 2019). This perception fosters feelings of interpersonal care and emotional connection, which are central to affective trust (Aboelenien et al., 2023). Furthermore, such perceived benevolence can deepen parasocial closeness and emotional security in live commerce settings. As consumers come to believe that the streamer will not exploit their reliance on recommendations, they develop stronger affective trust.

**H2a.**  *Ethics is positively associated with cognitive trust.*

**H2b.**  *Ethics is positively associated with affective trust.*

#### 3.1.3. Homophily

Homophily refers to the perceived similarity between an influencer and customers across dimensions such as attitudes, lifestyle, communication style, values, and consumption preferences (J. C. McCroskey et al., 1975; Shehzala et al., 2024). In the context of live commerce, homophily captures the extent to which consumers perceive the influencer as sharing their identity, tastes, and social world. Homophily was selected because perceived similarity is particularly important in the relationship between influencers and consumers; consumers often rely on shared values, preferences, and consumption experiences to judge whether influencers understand them and provide advice that is relevant to them. Such similarity can serve as a cognitive basis for evaluating influencers’ recommendations, and the study by Chatterjee et al. (2026) also confirmed that homophily can influence affective trust. Therefore, homophily is particularly important for both forms of trust. Such perceived similarity facilitates smoother and more resonant communication, thereby enhancing the persuasiveness of the streamer’s product recommendations (Shoenberger & Kim, 2023). Homophily is expected to promote cognitive trust because perceived similarity encourages customers to infer that the influencer relies on evaluative standards and decision criteria similar to their own. Consequently, the influencer’s product judgments are perceived as more relevant and diagnostic for consumers’ purchase decisions. When consumers believe that a similar other has evaluated a product in a way comparable to how they themselves would evaluate it, they are more likely to regard the streamer’s recommendations as competent and reliable guidance. Homophily is also expected to promote affective trust because homophily fosters interpersonal liking and a sense of shared identity, or “we-ness.” This reduces psychological distance and strengthens the parasocial closeness and emotional attachment that form the basis of affective trust (Sun et al., 2019; Wongkitrungrueng & Assarut, 2020). As consumers come to perceive the influencer as “someone like me” who understands their concerns and preferences, they are more likely to develop the emotional security.

**H3a.**  *Homophily is positively associated with cognitive trust.*

**H3b.**  *Homophily is positively associated with affective trust.*

### 3.2. Short-Form Video Characteristics

#### 3.2.1. Authenticity

Authenticity in live commerce refers to customers’ perception that an influencer’s intentions are sincere, that product-related claims are truthful. The influencer’s on-screen self-presentation reflects a real identity rather than a commercially constructed persona (M. Kim & Baek, 2025; Wongkitrungrueng & Assarut, 2020). Authentic influencers are characterized by honest product portrayals, including candid disclosure of product limitations, and consistency between promotional messages and personal usage experience. Authenticity would enhance cognitive trust because it functions as a diagnostic cue of the influencer’s integrity and reliability (Shehzala et al., 2024). When customers perceive that an influencer refrains from exaggeration and provides balanced, evidence-based information, they are more likely to infer that the influencer is a competent and dependable source of purchase-relevant judgments. Authenticity may improve affective trust because an influencer’s unfiltered emotional expression signals genuine care for customers rather than purely commercial motives (Shoenberger & Kim, 2023; Zhao & Kim, 2026). This reduces psychological distance and fosters the parasocial closeness and emotional attachment that underpin affective trust in live commerce (Chen et al., 2024; Sun et al., 2019).

**H4a.**  *Authenticity is positively associated with cognitive trust.*

**H4b.**  *Authenticity is positively associated with affective trust.*

#### 3.2.2. Originality

Originality refers to the degree to which an influencer’s live commerce content is perceived as novel, innovative, and distinct from that of other influencers on the platform (Shehzala et al., 2024). Original content is characterized by distinctive visual and narrative styles, creative program formats, and fresh thematic perspectives that differentiate the influencer within an increasingly homogeneous live commerce environment (Rodrigues et al., 2024). Originality is expected to foster cognitive trust because creating original content requires domain expertise, creative capability, and sustained effort. Customers may interpret such content as observable evidence that the influencer possesses the ability and diligence needed to provide reliable product information (Rodrigues et al., 2024). Originality is also expected to foster affective trust because original content reveals a distinctive personality and personal style.

**H5a.**  *Originality is positively associated with cognitive trust.*

**H5b.**  *Originality is positively associated with affective trust.*

#### 3.2.3. Entertainment

Entertainment refers to the degree to which live commerce video content provides enjoyment, fun, and recreational value for customers (Wang, 2020). Across media contexts, entertainment has long been recognized as a driver of positive affect and sustained audience engagement. In live commerce, entertainment is typically created through humor, engaging storytelling, music, visual spectacle, and playful real-time interaction with customers. Delivering enjoyable content requires communicative competence, presentational skill, and careful preparation. Customers may interpret these qualities as evidence of the influencer’s professionalism and ability, which are key foundations of cognitive trust (M. Kim & Baek, 2025; Wang, 2020). Enjoyable content elicits positive affect that customers may come to associate with the influencer as its source (Chen et al., 2024). In addition, humor and playful interaction can reduce psychological distance between the influencer and customers, thereby strengthening parasocial closeness and emotional attachment (Sun et al., 2019; Wongkitrungrueng & Assarut, 2020).

**H6a.**  *Entertainment is positively associated with cognitive trust.*

**H6b.**  *Entertainment is positively associated with affective trust.*

### 3.3. Cognitive Trust and Affective Trust

Consumer’s imitation intention refers to a customer’s willingness to follow an influencer’s product choices, usage behaviors, and consumption style (Ampornklinkaew, 2025). In live commerce, trust plays a central role in shaping such intention because customers often rely on influencers as important reference figures in evaluating products and making purchase decisions (B. Kim et al., 2023; Li et al., 2025). Cognitive trust is expected to be positively associated with consumer imitation intention because it is based on perceptions of the influencer’s competence, expertise, and reliability. When customers believe that an influencer provides accurate evaluations and dependable purchase-related judgments, they are more likely to regard the influencer as a credible model for their own consumption decisions. Under such conditions, imitating the influencer’s behavior becomes a rational way to reduce uncertainty and improve decision quality. Therefore, cognitive trust is likely to increase consumer imitation intention. Moreover, cognitive trust may provide a foundation for the development of affective trust, as consumers’ rational confidence in an influencer’s reliability and dependability can facilitate the emergence of stronger emotional bonds and interpersonal confidence (McAllister, 1995). Affective trust is also expected to be positively associated with consumer imitation intention because it is rooted in emotional attachment, interpersonal liking, and psychological closeness. When customers feel emotionally bonded with an influencer, they are more likely to identify with that person and adopt the influencer’s preferences, product choices, and consumption style. In this sense, imitation is not only a functional response but also a relational expression of affinity and symbolic closeness. Therefore, affective trust is likely to increase consumer imitation intention. Li et al. (2025) revealed the both cognitive and emotional trust play an important role in customer’s purchasing intention in live streaming shopping.

**H7.**  *Cognitive trust is positively associated with affective trust.*

**H8a.**  *Cognitive trust is positively associated with consumer imitation intention.*

**H8b.**  *Affective trust is positively associated with consumer imitation intention.*

## 4. Materials and Methods

### 4.1. Measurement Instrument

All constructs were measured using established multi-item scales adapted from the literature and modified to reflect the short-form live commerce context. Influencer social attractiveness was measured with three items adapted from L. L. McCroskey et al. (2006). Influencer ethics was measured with three items adapted from Awasthi and Singhal (2014) and Singh et al. (2012). Influencer homophily was measured with four items adapted from Ladhari et al. (2020). Video authenticity was measured with four items adapted from Kusbianto et al. (2025). Video originality was measured with three items adapted from Rodrigues et al. (2024). Video entertainment was measured with three items adapted from Wang (2020). Cognitive trust and affective trust were measured using items adapted from McAllister (1995). Imitation intention was measured with four items adapted from Belanche et al. (2021). All items were scored on a seven-point Likert scale ranging from 1 (strongly disagree) to 7 (strongly agree). The complete list of measurement items appears in Table 1.

### 4.2. Data Collection and Sample

Data were collected in August 2026 via the Chinese online survey platform Wenjuanxing. The target population comprised consumers who had watched influencer short-form videos and influencer live commerce broadcasts on Chinese short-form video platforms. A screening question confirmed platform usage and live commerce viewership experience. Respondents accessing the survey from platforms other than TikTok (Douyin), Kuaishou, or Xiaohongshu were excluded. A total of 306 usable responses were retained after removing cases with missing data, straight-line responding, and implausible response times.

Table 2 presents the demographic profile of the sample. The sample was approximately gender-balanced (57.84% female). The most represented age group was 20–30 years (35.29%), consistent with the predominantly young user base of short-form video platforms in China. TikTok (Douyin) was the most used platform (42.48%), followed by Xiaohongshu (30.39%) and Kuaishou (27.12%). The largest proportion of respondents (33.33%) reported monthly household incomes lower than RMB 5000.

### 4.3. Analytical Approach

The data were analyzed using PLS-SEM implemented in SmartPLS 4.0. PLS-SEM is appropriate for this study because the research model is complex, includes multiple reflective constructs, and the research goal is predictive in addition to confirmatory (Garg & Bakshi, 2024; Hair et al., 2019). The analysis proceeded in two stages. First, the measurement model was evaluated for reliability and validity, factor loadings, and the Heterotrait–Monotrait (HTMT) criterion. Second, the structural model was tested through bootstrapping with 5000 resamples to obtain robust estimates of path coefficients and indirect effects.

## 5. Results

### 5.1. Measurement Model

Table 3 reports the reliability and validity statistics for all constructs. Cronbach’s α values ranged from 0.896 to 0.939, all well above the 0.70 threshold. Composite reliability (CR) values ranged from 0.935 to 0.956, exceeding the 0.70 criterion (Fornell & Larcker, 1981). The average variance extracted (AVE) values ranged from 0.824 to 0.848, all exceeding the 0.50 threshold, confirming convergent validity. All factor loadings exceeded 0.70, with values ranging from 0.891 to 0.931. As shown in Table 4, discriminant validity was first assessed using the Fornell–Larcker criterion. For every construct, the square root of its AVE exceeded its correlations with all other constructs, confirming discriminant validity. Discriminant validity was further confirmed using the HTMT ratio in Table 5. All HTMT values remained below the conservative 0.85 threshold (Henseler et al., 2015), with the highest observed value occurring between social attractiveness and ethics. Additionally, to assess multicollinearity among the predictor constructs, variance inflation factors (VIFs) were examined for all inner model paths. All VIF values ranged from 1.000 to 2.231, well below the recommended threshold of 5.0 (Kock, 2015), indicating that multicollinearity was not a concern in the structural model.

### 5.2. Structural Model and Hypothesis Testing

**Figure 2:**
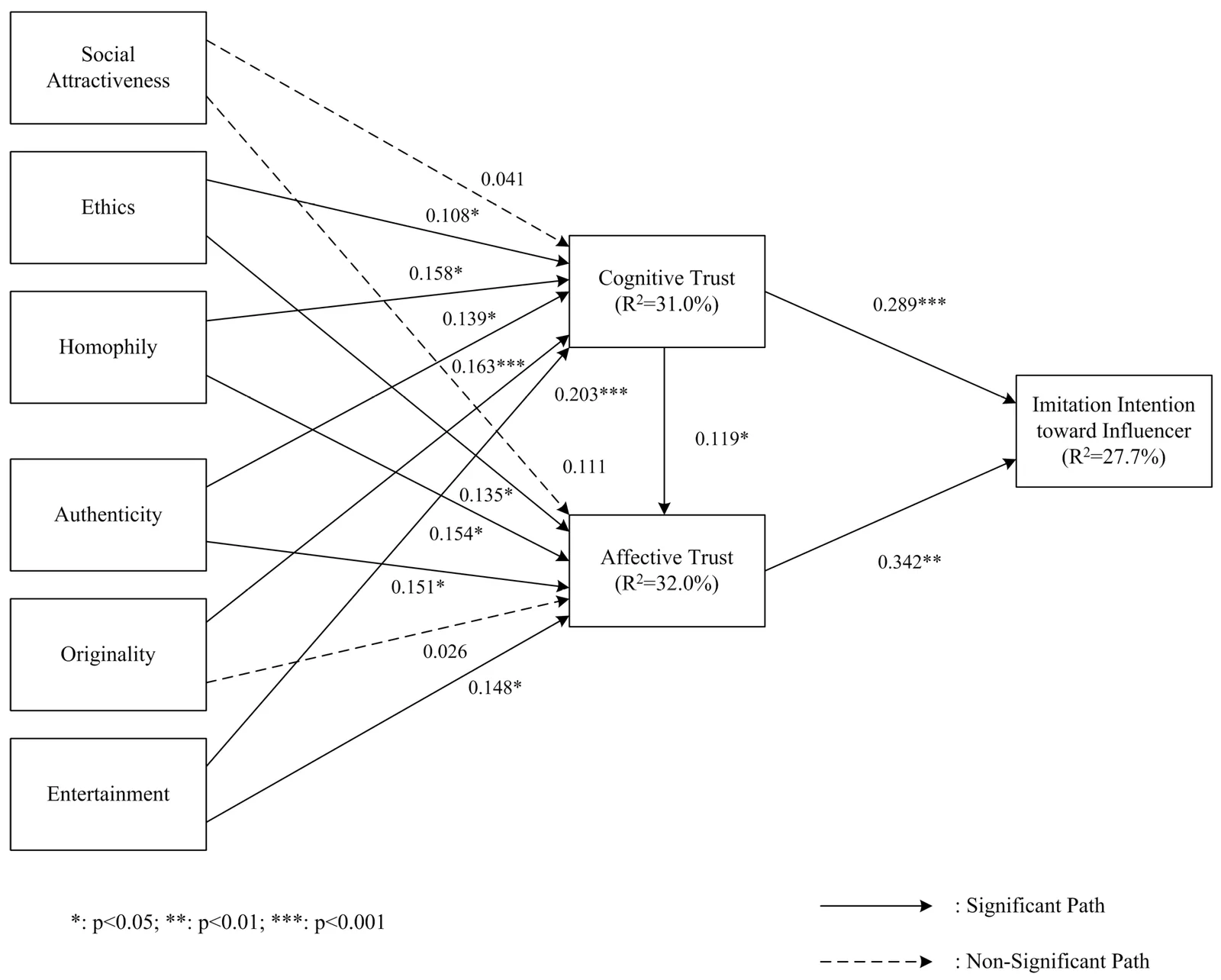
Structural model results.

Table 6 reports the structural model results based on bootstrapping with 5000 resamples. Figure 2 illustrates the path model with standardized path coefficients.

The results indicate that cognitive trust is significantly related to both influencer characteristics and short-form video attributes. Specifically, ethics, homophily, authenticity, originality, and entertainment had significant positive effects on cognitive trust. Accordingly, H2a, H3a, H4a, H5a, and H6a were supported. However, social attractiveness did not significantly influence cognitive trust; thus, H1a was not supported. By contrast, affective trust was significantly influenced by ethics, homophily, authenticity, and entertainment, supporting H2b, H3b, H4b, and H6b. However, social attractiveness and originality did not significantly influence affective trust. Therefore, H1b and H5b were not supported. In addition, cognitive trust had a significant positive effect on affective trust, supporting H7. Regarding the effects of the organismic states on the behavioral response, both cognitive trust and affective trust significantly influenced consumers’ imitation intention toward influencers. Thus, H8a and H8b were supported.

The *R*^2^ values for endogenous variables were as follows: cognitive trust = 0.310, affective trust = 0.320, and imitation intention = 0.277. Cognitive trust demonstrated the highest explanatory power, consistent with its role as the primary mediator of stimulus-to-intention pathways.

### 5.3. Predictive Validity and Effect Sizes

PLSpredict analysis confirmed the predictive validity of the model. All Q^2^predict values for endogenous indicator variables were positive, ranging from 0.168 to 0.242, confirming predictive relevance. Critically, the PLS-SEM root mean square error was lower than the linear model RMSE for all indicators, demonstrating that the proposed model has stronger out-of-sample predictive power than a naïve linear benchmark. Effect size analysis (*f*^2^) revealed that most paths exhibited small effect sizes, with f^2^ values ranging from 0.001 to 0.138 (Cohen, 1988). The largest effect sizes were observed for affective trust and cognitive trust on imitation intention (*f*^2^ = 0.138 and 0.098, respectively), indicating that these two trust dimensions are the most practically relevant predictors of consumers’ imitation intention.

### 5.4. Mediation Analysis

Mediation analysis was conducted using bootstrapped indirect effects to examine whether cognitive trust and affective trust transmitted the effects of influencer characteristics and short-form video attributes to consumers’ imitation intention toward influencers. The significance of the indirect effects was assessed using bias-corrected 95% bootstrap confidence intervals based on 5000 bootstrap resamples, with an indirect effect considered significant when the confidence interval did not include zero. Table 7 presents the results of the mediation analysis.

The results indicate that cognitive trust significantly mediated five of the six stimulus-to-imitation relationships. Specifically, the indirect effects of ethics, homophily, authenticity, originality, and entertainment on imitation intention through cognitive trust were significant, whereas the indirect effect of social attractiveness through cognitive trust was not significant. These findings indicate that cognitive trust serves as an important transmission mechanism through which most influencer-related and content-related cues influence consumers’ imitation intention.

Affective trust significantly mediated four of the six stimulus-to-imitation relationships. Specifically, the indirect effects of ethics, homophily, authenticity, and entertainment on imitation intention through affective trust were significant, whereas the indirect effects of social attractiveness and originality were not significant. In addition, the sequential indirect effects through cognitive trust and affective trust were significant for ethics, homophily, authenticity, originality, and entertainment, whereas the sequential indirect effect of social attractiveness was not significant. The indirect effect of cognitive trust on imitation intention through affective trust was also significant, indicating that cognitive trust can further influence imitation intention by contributing to the development of affective trust.

Taken together, the mediation results demonstrate both parallel and sequential trust mechanisms. Cognitive trust independently mediated the effects of ethics, homophily, authenticity, originality, and entertainment, whereas affective trust independently mediated the effects of ethics, homophily, authenticity, and entertainment. Moreover, except for social attractiveness, the antecedent variables also influenced imitation intention through the sequential pathway of cognitive trust and affective trust. These findings suggest that cognitive and affective trust operate not only as distinct but complementary mediating mechanisms, but also as a sequential trust process through which influencer- and content-related characteristics are translated into consumers’ imitation intention in short-form live commerce.

Common method bias (CMB) was assessed using the full collinearity approach. As shown in Table 8, The full collinearity VIF values for all constructs ranged from 1.277 to 1.534, well below the recommended threshold of 3.3. Therefore, the results indicate that common method bias was not a serious concern in this study.

## 6. Discussion

### 6.1. Summary of Findings

This study uses the S-O-R framework as the overall organizing framework to examine how influencer characteristics and short-form video content characteristics affect consumers’ imitation intention through cognitive trust and affective trust in the context of live commerce. The results show that different stimulus factors do not exert consistent effects on the two types of trust but instead exhibit clearly differentiated “stimulus–psychological state–behavioral response” relationships. The model explains 31.0% of the variance in cognitive trust, 32.0% of the variance in affective trust, and 27.7% of the variance in imitation intention.

Regarding trust formation, ethics, homophily, authenticity, and entertainment significantly enhance both cognitive trust and affective trust. In contrast, originality significantly enhances only cognitive trust, while its effect on affective trust is not significant. Among these factors, entertainment has the strongest effect on cognitive trust, indicating that enjoyable and appealing content can provide important cues for consumers’ rational evaluations of influencers. Regarding affective trust, homophily has the strongest effect, indicating that similarity enhances consumers’ affective trust in influencers. This also suggests that consumers are more inclined to choose influencers who are similar to themselves and share the same values. Social attractiveness has no significant effect on either cognitive trust or affective trust. This indicates that when consumers evaluate influencers simultaneously, merely perceiving an influencer as approachable or easy to get along with may not be sufficient to form genuine trust. Meanwhile, the insignificant effect of originality on affective trust also indicates that although novel and distinctive content can enhance consumers’ rational judgments of an influencer’s competence, information value, and expertise, it does not necessarily further generate feelings of being cared for, emotional security, or relational attachment.

Furthermore, cognitive trust had a significant positive effect on affective trust, indicating that consumers’ rational evaluations of an influencer’s reliability and competence may provide a foundation for the formation of affective trust. The mediation analysis further revealed differentiated parallel and sequential trust mechanisms. Cognitive trust significantly mediated the effects of ethics, homophily, authenticity, originality, and entertainment on imitation intention, whereas the indirect effect of social attractiveness through cognitive trust was not significant. Affective trust significantly mediated the effects of ethics, homophily, authenticity, and entertainment, while the indirect effects of social attractiveness and originality through affective trust were not significant. More importantly, the indirect effect of cognitive trust on imitation intention through affective trust was significant. At the antecedent level, ethics, homophily, authenticity, originality, and entertainment also significantly influenced imitation intention through the sequential pathway of cognitive trust and affective trust, whereas the sequential indirect effect of social attractiveness was not significant. These findings indicate that cognitive and affective trust operate both as distinct mediating mechanisms and, for most antecedents, as a sequential trust process through which external cues are translated into imitation intention.

Finally, both cognitive trust and affective trust significantly enhanced consumers’ imitation intention, with affective trust exerting the stronger direct effect. This result indicates that, in influencer-driven live commerce, consumers’ imitation of influencers does not depend solely on rational confidence in influencers’ expertise, reliability, and judgment. Emotional security, perceived care, and relational connection also play an important role in determining whether consumers regard influencers as behavioral reference models. From the perspective of social learning theory, trust helps consumers move beyond merely observing influencers to viewing them as behavioral models whose consumption-related choices, suggestions, and recommendations are worthy of adoption. Together with the significant sequential mediation results, this finding further suggests that rational confidence in an influencer may facilitate emotional trust, which in turn strengthens consumers’ willingness to follow the influencer’s consumption-related behavior.

### 6.2. Theoretical Contributions

This study makes the following theoretical contributions to influencer marketing and live commerce research. First, this study does not regard the extension of the S-O-R framework itself as a theoretical contribution but instead uses it as the overall framework for organizing the research model to distinguish between external influencer and content stimuli, consumers’ internal psychological states of trust, and imitation intention as a behavioral response. The results show that different stimuli do not affect consumers’ internal psychological states in the same way. Ethics, homophily, authenticity, and entertainment simultaneously affect cognitive trust and affective trust; originality affects only cognitive trust but does not significantly affect affective trust; and social attractiveness has no significant effect on either type of trust. These differentiated results indicate that trust formation in live commerce does not arise from consumers’ generally positive evaluations of influencers or content but depends on the specific informational and relational meanings conveyed by different stimuli.

Second, by simultaneously examining the differentiated roles of cognitive trust and affective trust and the relationship between them, this study deepens the application of the dual-trust perspective in influencer–consumer relationships. Consistent with McAllister (1995), cognitive trust significantly influences affective trust, indicating that rational assessments of an influencer’s reliability and competence can provide a foundation for emotional trust. More importantly, the significant indirect effect of cognitive trust on imitation intention through affective trust demonstrates that the two trust dimensions are not merely parallel psychological states. Cognitive trust can contribute to imitation intention both directly and indirectly through affective trust. In addition, the significant sequential indirect effects for ethics, homophily, authenticity, originality, and entertainment show that, for most external cues, rational trust can serve as an intermediate foundation through which affective trust is subsequently formed and translated into imitation intention. Thus, the findings provide a more nuanced dual-trust mechanism that combines differentiated parallel pathways with a theoretically meaningful sequential process.

Third, this study extends influencer marketing research from transaction-oriented outcomes represented by purchase intention to influencer-centered imitation intention. Drawing on social learning theory, the findings show that both cognitive and affective trust help explain why consumers regard influencers as behavioral reference models and become willing to adopt or follow their consumption choices, suggestions, and recommendations. Affective trust exerted a stronger direct effect on imitation intention than cognitive trust, highlighting the importance of emotional security and relational connection in influencer-directed imitation. At the same time, the significant indirect pathway from cognitive trust through affective trust to imitation intention indicates that rational confidence can also facilitate imitation indirectly by fostering emotional trust. Thus, the study identifies trust not merely as a predictor of transactional decisions but as a psychological mechanism through which observed influencers become meaningful behavioral reference models.

Finally, the mediation findings reveal that influencer- and content-related characteristics can operate through both differentiated and sequential trust mechanisms. Ethics, homophily, authenticity, and entertainment exerted significant indirect effects through both cognitive and affective trust, whereas originality exerted a significant indirect effect through cognitive trust but not through affective trust alone. However, originality also produced a significant sequential indirect effect through cognitive trust and affective trust. This pattern suggests that originality may not directly generate affective trust, but may first strengthen rational confidence in the influencer, which subsequently contributes to affective trust and imitation intention. More broadly, the significant sequential indirect effects for five of the six antecedents demonstrate that different external cues can be translated into imitation intention through a cognitive-to-affective trust process, while social attractiveness remains insufficient to activate either pathway. These findings clarify how different influencer and content cues enter the trust-building process at different psychological stages.

### 6.3. Practical Implications

First, the selection of influencers should not rely solely on their external attractiveness or approachability but should place greater emphasis on homophily between influencers and target consumers, as well as influencers’ ethical behavior. This study found that social attractiveness had no significant effect on either cognitive trust or affective trust, whereas homophily significantly enhanced both types of trust and had the strongest effect on affective trust. This indicates that, for brands and platforms engaged in live commerce, simply selecting influencers who are highly approachable or easy to relate to may not be sufficient to establish consumer trust. Although influencers’ external attractiveness can attract consumers, attractiveness alone is not sufficient for building long-term trust. In contrast, greater attention should be paid to the degree of fit between influencers and target consumers in terms of values, lifestyles, interests, and consumption preferences. Influencers themselves can also share their personal experiences, consumption habits, and values, making it easier for consumers to perceive commonalities between themselves and the influencers, thereby strengthening relational connections. At the same time, the significant effect of ethics on trust indicates that influencers should emphasize honesty and responsibility in their recommendation behaviors, such as truthfully communicating their product experiences, avoiding exaggerated claims about product effects, and clearly disclosing commercial partnerships in order to maintain consumers’ long-term trust.

Second, in terms of short-form video content strategies, influencers need to recognize that authenticity, originality, and entertainment play different roles in the formation of trust. This study found that authenticity and entertainment can simultaneously enhance cognitive trust and affective trust, with entertainment having the strongest effect on cognitive trust. Therefore, influencers should not regard entertaining content merely as a tool for attracting traffic and increasing views but should present product information in appealing and enjoyable ways so that it can be more easily accepted and understood by consumers. At the same time, the important role of authenticity indicates that influencers should reduce overly commercialized or deliberately packaged content and enhance consumers’ trust in both the content and the influencer through authentic product-use processes, personal experiences, and more natural forms of expression. On the other hand, originality significantly affects only cognitive trust and has no significant effect on affective trust. This means that distinctive, novel, and creative content is more suitable for strengthening consumers’ judgments of influencers’ competence, expertise, and information value, but relying solely on content innovation is insufficient to establish emotional connections between consumers and influencers. Therefore, while pursuing content differentiation, influencers also need to combine authenticity with relational communication to develop consumers’ affective trust.

Third, influencers, live commerce platforms, and companies should extend trust management from merely emphasizing expertise and reliability to building emotional relationships between consumers and influencers. This study found that both cognitive trust and affective trust significantly enhance imitation intention, but affective trust has a stronger effect. This indicates that if the goal of live commerce is not only to encourage consumers to purchase a single product but also to encourage them to continuously accept influencers’ consumption suggestions and recommendations, merely demonstrating influencers’ expertise and reliability is insufficient. Influencers also need to strengthen consumers’ sense of emotional security and relational connections through continuous interaction, responding to consumer comments, sharing personal experiences, and paying attention to consumer needs, so that consumers gradually regard influencers as trustworthy behavioral reference models. Particularly for influencers who build their personal brands over the long term on short-form video platforms, such a relationship-oriented strategy can enable their influence to go beyond individual product transactions and make consumers more willing to continuously refer to and follow their consumption choices, suggestions, and recommendations. For platforms and companies, attention should not be focused solely on short-term sales conversion; they should also develop influencers into trustworthy behavioral reference models for consumers, thereby creating more stable and sustained influence on consumer behavior.

### 6.4. Limitations and Future Research

Several limitations of this study provide opportunities for future research. First, this study employed a cross-sectional survey design, which limits the ability to establish the temporal and causal ordering among influencer- and content-related characteristics, trust, and imitation intention. In particular, reverse causality cannot be ruled out. Consumers who already regard an influencer as a behavioral reference model or have a stronger desire to imitate that influencer may subsequently perceive the influencer as more similar, authentic, ethical, or trustworthy. Therefore, the observed relationships should be interpreted as associations rather than definitive causal effects. Future research could employ longitudinal or experimental designs to examine the temporal ordering among influencer and content perceptions, cognitive and affective trust, and imitation intention.

Second, this study focused on six antecedents representing influencer-related characteristics (social attractiveness, ethics, and homophily) and content-related characteristics (authenticity, originality, and entertainment). Although these variables capture two complementary sources of trust formation, they do not exhaust all possible determinants of cognition-based and affective trust. Other factors, such as expertise, perceived credibility, parasocial interaction, perceived usefulness, social presence, and product–influencer fit, may also influence consumers’ trust in influencers. Future research could incorporate these variables or examine them as boundary conditions to provide a more comprehensive explanation of trust formation in live commerce.

Third, the relatively small sample size may limit the statistical power and generalizability of the findings. Although the sample was sufficient for the PLS-SEM analysis conducted in this study, a larger and more diverse sample would provide more stable parameter estimates and allow for more detailed subgroup comparisons. Future studies should therefore replicate the model using larger samples and respondents with more diverse demographic and usage characteristics.

Finally, the findings should be interpreted within the specific context of short-form video and live commerce. Consumer responses to influencers may vary across platforms, product categories, cultural settings, and levels of consumer involvement. Future research could conduct cross-platform, cross-product, or cross-cultural comparisons to examine whether the relative roles of cognition-based and affective trust differ across contexts. In addition, although the present study identified significant sequential indirect effects through cognitive and affective trust for five of the six antecedents, future research could examine boundary conditions such as relationship duration, social presence, or relationship proneness to determine when this cognitive-to-affective trust process becomes stronger or weaker.

## Figures and Tables

**Table 1:** Measurement items and sources.

Construct	Item	Mean	AVE
Social Attractiveness	SA1	I think this influencer could be a friend of mine.	L. L. McCroskey et al. (2006)
	SA2	I think this influencer is someone I would talking with.	
	SA3	I think this influencer would be pleasant to be with.	
Ethics	ETH1	This influencer upholds high ethical standards.	Awasthi and Singhal (2014); Singh et al. (2012)
	ETH2	This influencer is ethical.	
	ETH3	This influencer is responsible toward their followers.	
Homophily	HOM1	I have a lot in common with this influencer.	Ladhari et al. (2020)
	HOM2	This influencer and I resemble each other in many ways.	
	HOM3	This influencer thinks similarly to me.	
	HOM4	This influencer and I share the same values.	
Authenticity	AUTH1	I believe the video content is objective and factual.	Kusbianto et al. (2025)
	AUTH2	The video portrayal matches the actual product characteristics.	
	AUTH3	The influencer’s behavior, expression, and speech in the video appear natural and genuine.	
	AUTH4	The influencer’s personal style and image in the video feel real.	
Originality	ORI1	The video showcases the influencer’s distinctive style.	Rodrigues et al. (2024)
	ORI2	The format or topic of the video is novel.	
	ORI3	The video provided me with a fresh viewing experience.	
Entertainment	ENT1	I find watching this influencer’s videos highly entertaining.	Wang (2020)
	ENT2	The videos help me relax and enjoy my leisure time.	
	ENT3	Watching the videos helps me temporarily forget my worries.	
Cognitive Trust	CT1	I believe this influencer provides advice based on solid knowledge and expertise in their field.	McAllister (1995)
	CT2	I believe this influencer will act responsibly regarding the information they share and honor their commitments to followers.	
	CT3	I can rely on this influencer not to mislead me or cause inconvenience through careless or unverified recommendations.	
	CT4	Most people, even those who are not loyal fans, respect and trust this influencer’s capability and recommendations.	
Affective Trust	AT1	In any situation, this influencer is ready to provide help and support.	McAllister (1995)
	AT2	When interacting, this influencer cares about my interests.	
	AT3	I trust this influencer because they genuinely care about their users.	
Imitation Intention	II1	I would feel comfortable trying products or consumption choices presented by this influencer	Belanche et al. (2021)
	II2	I would not hesitate to take into account the consumption suggestions provided by this influencer.	
	II3	I would feel comfortable following the consumption suggestions provided by this influencer.	
	II4	I would rely on the consumption recommendations provided by this influencer	

**Table 2:** Sample demographic characteristics (n = 306).

Variable	Category	n	Percent
Gender	Female	177	57.84
	Male	129	42.16
Age	20–30	108	35.29
	31–40	93	30.39
	41–50	68	22.22
	≥51	37	12.09
Education	High school or below	32	10.46
	Associate degree	119	38.89
	Bachelor’s degree	116	37.91
	Master’s degree	26	8.50
	Doctoral degree	13	4.25
Primary platform	TikTok (Douyin)	130	42.48
	Xiaohongshu	93	30.39
	Kuaishou	83	27.12
Monthly household income	<5000	102	33.33
	5000–10,000	86	28.10
	10,001–20,000	57	18.63
	20,001–50,000	44	14.38
	>50,000	17	5.56

**Table 3:** Reliability and validity statistics.

Construct	Item	Mean	Standard Deviation	Factor Loading	Cronbach’s α	CR	AVE
Social Attractiveness	SA1	5.49	1.756	0.912	0.907	0.941	0.843
	SA2	5.42	1.717	0.925			
	SA3	5.49	1.652	0.917			
Ethics	ETH1	5.45	1.576	0.931	0.907	0.942	0.843
	ETH2	5.44	1.679	0.923			
	ETH3	5.38	1.783	0.901			
Homophily	HOM1	5.37	1.678	0.924	0.939	0.956	0.844
	HOM2	5.36	1.793	0.921			
	HOM3	5.35	1.740	0.928			
	HOM4	5.39	1.709	0.902			
Authenticity	AUTH1	5.65	1.636	0.891	0.929	0.949	0.824
	AUTH2	5.57	1.567	0.926			
	AUTH3	5.53	1.601	0.895			
	AUTH4	5.51	1.699	0.917			
Originality	ORI1	5.4	1.692	0.929	0.911	0.944	0.848
	ORI2	5.42	1.626	0.909			
	ORI3	5.47	1.662	0.925			
Entertainment	ENT1	5.39	1.654	0.911	0.898	0.936	0.831
	ENT2	5.47	1.689	0.918			
	ENT3	5.39	1.560	0.906			
Cognitive Trust	CT1	5.54	1.735	0.915	0.929	0.950	0.825
	CT2	5.4	1.661	0.904			
	CT3	5.37	1.835	0.899			
	CT4	5.47	1.748	0.916			
Affective Trust	AT1	5.3	1.745	0.906	0.896	0.935	0.827
	AT2	5.48	1.691	0.906			
	AT3	5.48	1.782	0.918			
Imitation Intention	II1	5.48	1.654	0.920	0.933	0.952	0.832
	II2	5.48	1.75	0.913			
	II3	5.57	1.707	0.920			
	II4	5.46	1.634	0.896			

**Table 4:** Discriminant validity: Fornell–Larcker criteria.

	1	2	3	4	5	6	7	8	9
1. Social Attractiveness	0.918								
2. Ethics	0.230	0.918							
3. Homophily	0.318	0.324	0.919						
4. Authenticity	0.293	0.300	0.454	0.907					
5. Originality	0.292	0.323	0.360	0.368	0.921				
6. Entertainment	0.372	0.333	0.347	0.363	0.307	0.912			
7. Cognitive Trust	0.280	0.331	0.399	0.389	0.381	0.410	0.908		
8. Affective Trust	0.331	0.352	0.409	0.403	0.303	0.399	0.385	0.910	
9. Imitation Intention	0.275	0.296	0.384	0.379	0.281	0.400	0.421	0.453	0.912

**Table 5:** Discriminant validity: Heterotrait–Monotrait ratio.

	1	2	3	4	5	6	7	8	9
1. Social Attractiveness									
2. Ethics	0.252								
3. Homophily	0.343	0.348							
4. Authenticity	0.318	0.326	0.483						
5. Originality	0.321	0.355	0.387	0.399					
6. Entertainment	0.412	0.368	0.376	0.396	0.340				
7. Cognitive Trust	0.304	0.360	0.426	0.417	0.411	0.448			
8. Affective Trust	0.366	0.389	0.445	0.440	0.333	0.444	0.422		
9. Imitation Intention	0.296	0.321	0.410	0.405	0.304	0.435	0.449	0.495	

**Table 6:** Structural model results.

Hypothesis	Cause	Effect	Path Coefficient	Standard Deviation	*t* Statistics	*p* Values	Result
H1a	Social Attractiveness	Cognitive Trust	0.041	0.052	0.790	0.429	Not Supported
H1b	Social Attractiveness	Affective Trust	0.111	0.064	1.744	0.081	Not Supported
H2a	Ethics	Cognitive Trust	0.108	0.055	1.984	0.047	Supported
H2b	Ethics	Affective Trust	0.135	0.061	2.225	0.026	Supported
H3a	Homophily	Cognitive Trust	0.158	0.065	2.423	0.015	Supported
H3b	Homophily	Affective Trust	0.154	0.061	2.514	0.012	Supported
H4a	Authenticity	Cognitive Trust	0.139	0.054	2.552	0.011	Supported
H4b	Authenticity	Affective Trust	0.151	0.064	2.344	0.019	Supported
H5a	Originality	Cognitive Trust	0.163	0.059	2.788	0.005	Supported
H5b	Originality	Affective Trust	0.026	0.054	0.476	0.634	Not Supported
H6a	Entertainment	Cognitive Trust	0.203	0.055	3.691	0.000	Supported
H6b	Entertainment	Affective Trust	0.148	0.060	2.477	0.013	Supported
H7	Cognitive Trust	Affective Trust	0.119	0.060	1.983	0.047	Supported
H8a	Cognitive Trust	Imitation Intention	0.289	0.061	4.760	0.000	Supported
H8b	Affective Trust	Imitation Intention	0.342	0.061	5.654	0.000	Supported

**Table 7:** Mediation analysis results.

Indirect Path	Path Coefficient	Standard Deviation	*t* Statistics	*p* Values	95% Bias-Corrected CI	Result
SA → CT → II	0.012	0.016	0.752	0.452	[−0.01521, 0.04689]	Not significant
SA → AT → II	0.038	0.022	1.695	0.09	[−0.00235, 0.08618]	Not significant
SA → CT → AT → II	0.002	0.002	0.672	0.501	[−0.00151, 0.00905]	Not significant
ETH → CT → II	0.031	0.018	1.784	0.074	[0.00370, 0.07405]	Significant
ETH → AT → II	0.046	0.023	2.026	0.043	[0.00823, 0.10020]	Significant
ETH → CT → AT → II	0.004	0.004	1.258	0.208	[0.00032, 0.01629]	Significant
HOM → CT → II	0.046	0.022	2.072	0.038	[0.01060, 0.09907]	Significant
HOM → AT → II	0.053	0.024	2.215	0.027	[0.01297, 0.10781]	Significant
HOM → CT → AT → II	0.006	0.005	1.292	0.197	[0.00044, 0.02259]	Significant
AUTH → CT → II	0.04	0.019	2.104	0.035	[0.01024, 0.08534]	Significant
AUTH → AT → II	0.052	0.025	2.045	0.041	[0.01032, 0.11083]	Significant
AUTH → CT → AT → II	0.006	0.004	1.484	0.138	[0.00074, 0.01753]	Significant
ORI → CT → II	0.047	0.020	2.389	0.017	[0.01601, 0.09514]	Significant
ORI → AT → II	0.009	0.019	0.470	0.638	[−0.02349, 0.05173]	Not significant
ORI → CT → AT → II	0.007	0.005	1.416	0.157	[0.00076, 0.02096]	Significant
ENT → CT → II	0.059	0.021	2.852	0.004	[0.02691, 0.11087]	Significant
ENT → AT → II	0.051	0.024	2.093	0.036	[0.01347, 0.10832]	Significant
ENT → CT → AT → II	0.008	0.005	1.611	0.107	[0.00119, 0.02273]	Significant
CT → AT → II	0.041	0.023	1.801	0.072	[0.00350, 0.09196]	Significant

Note: SA: Social attractiveness, ETH: Ethics, HOM: Homophily, AUTH: Authenticity, ORI: Originality, ENT: Entertainment, CT: Cognitive Trust, AT: Affective Trust, II: Imitation Intention.

**Table 8:** Common Method Bias Assessment.

Construct	*R* ^2^	Full Collinearity VIF
Social Attractiveness (SA)	0.217	1.277
Ethics (ETH)	0.227	1.294
Homophily (HOM)	0.336	1.506
Authenticity (AUTH)	0.330	1.493
Originality (ORI)	0.259	1.350
Entertainment (ENT)	0.329	1.490
Cognitive Trust (CT)	0.341	1.517
Affective Trust (AT)	0.348	1.534
Imitation Intention (II)	0.333	1.499

## Data Availability

Data is contained within the Supplementary Materials. Further inquiries can be directed to the corresponding author.

## Supplementary Materials

The following supporting information can be downloaded at: https://www.mdpi.com/article/10.3390/bs16091641/s1, File S1: Survey Data.
